# Helicity-dependent photocurrent induced by the in-plane transverse electric current in an InAs quantum well

**DOI:** 10.1038/srep31189

**Published:** 2016-08-09

**Authors:** J. B. Li, X. G. Wu, G. W. Wang, Y. Q. Xu, Z. C. Niu, X. H. Zhang

**Affiliations:** 1 State Key Laboratory of Superlattices and Microstructures, Institute of Semiconductors, Chinese Academy of Science, P. O. Box 912, Beijing 100083, P. R. China

## Abstract

We report the observation of a new type of helicity-dependent photocurrent induced by an in-plane transverse direct electric current in an InAs quantum well. The amplitude of the photocurrent depends linearly on the transverse current. Moreover, the observed incident azimuth-angle dependence of this photocurrent is different from that induced by the circular photogalvanic effect. This new photocurrent appears as a result of an asymmetrical carrier distribution in both the conduction and valence bands induced by the transverse current. The photoexcited carrier density created by interband transition processes is thus modulated and leads to the observed new azimuth-angle dependence. The observed efficient generation of the helicity-dependent photocurrent offers an effective approach to manipulate electron spins in two-dimensional semiconductor systems with the added advantage of electrical control of the spin-related photocurrent in spintronic applications.

Spin-related phenomena^1^,^2^,^3^,^4^,^5^,^6^,^7^ have drawn much attention after Datta and Das proposed the spin-field effect transistor^8^. Being closely connected to the spin of the carrier, the helicity-dependent photocurrent (HDPC)^4^,^9^,^10^,^11^,^12^,^13^,^14^,^15^,^16^,^17^,^18^ is known to be an efficient method to study the spin-orbit interaction, which is crucial in spin-based electronic devices. Among the different HDPCs, the spin photocurrent generated through the circular photogalvanic effect (CPGE) is most impressive^9^,^10^,^16^,^17^,^18^,^19^,^20^,^21^,^22^,^23^,^24^. The CPGE photocurrent is induced through an imbalance in spin population of the nonequilibrium carriers excited by the circularly polarised light as a result of the 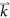-linear band splitting caused by the spin-orbit interaction and the optical selection rule. Here, 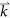 is the two-dimensional electron wave vector in the plane of the quantum well. Moreover, a homogeneous spin polarisation generated by any means was demonstrated to be able to generate a current if the structural symmetry allows a 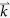-linear term in the Hamiltonian^9^. This phenomenon is known as the spin-galvanic effect (SGE)^4^. Ganichev *et al*.^23^,^24^ have presented a microscopic model to bridge the phenomenological (spinless) theory of the SGE (and the CPGE) and carrier spins, so that the effective Rashba and Dresselhaus spin-orbit coupling fields can be quantitatively investigated. Another type of HDPC generated under the excitation of circularly polarised light with an external magnetic field originates from the circular magnetogyrotropic photogalvanic effect^11^,^12^,^25^, which is one type of SGE resulting from the optical orientation of carriers, and subsequent Larmor precession of the oriented electron spins as well as the asymmetric spin-relaxation processes. The photo-induced anomalous Hall effect observed by Yu *et al*.^13^ can also induce a HDPC and is attributed to the lateral deflections of spin-polarised carriers generated by the circularly polarised light due to the spin Hall effect. Recently, Ma *et al*.^14^ have reported observing photo-assisted generation of HDPC. They suggest that this HDPC originates from the spin-orbit coupling as well as CPGE. Additionally, the optical field and magnetic field applied in manipulating the HDPC, an electric field or an electric current, may also enable the HDPC to be modulate. However, little has been reported so far on using the transverse electric current to manipulate the HDPC.

In this study, a new type of HDPC induced by an in-plane transverse direct current (here after referred to as the transverse current) is observed in an InAs quantum well. The amplitude of the observed HPDC shows a linear dependence on the transverse current. In addition, in contrast to the photocurrent originating purely from the circular photogalvanic effect, the transverse-current-driven HPDC response is observed to exhibit a different dependence on the incident azimuth angle. In the present paper, the imbalanced distribution of spin-polarised carriers including holes and electrons in momentum space is discussed, and the asymmetrical distribution of photoexcited electron density caused by the transverse current is suggested to be responsible for the observed HPDC. The observed efficient generation of the HDPC by the direct electric current offers an effective approach to manipulate carrier spins in two-dimensional semiconductor systems, which can be a great advantage for the electric control of spin-related photocurrent for the future spintronic applications.

## Results

### Sample preparation and experimental details

**Figure 1:**
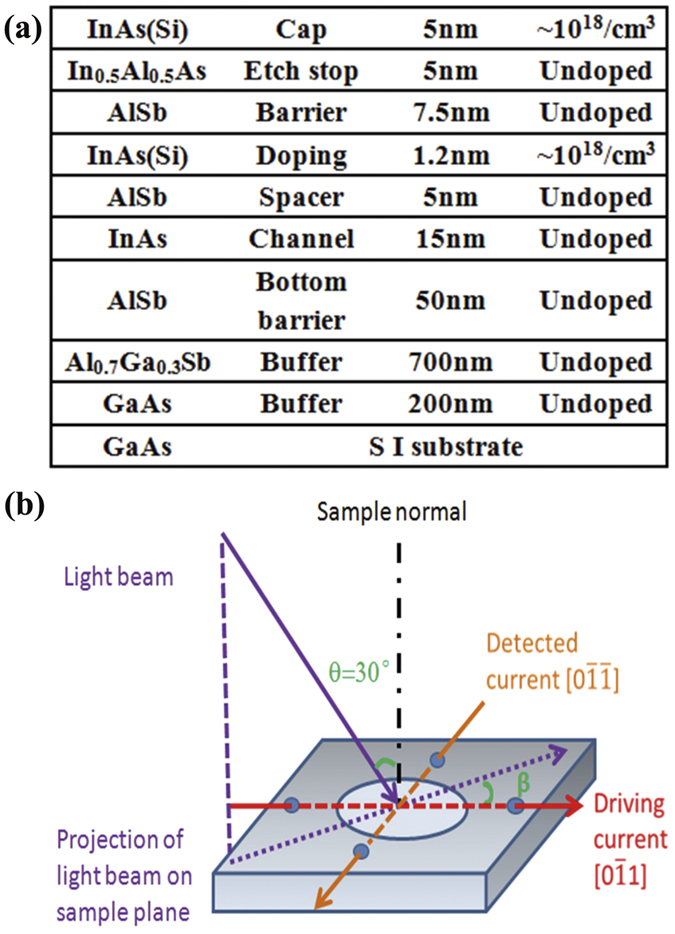
(**a**) Sketch of the InAs quantum well structure; (**b**) schematic of the experimental set up, the incident azimuth angle *β* is the angle of the incident plane of the excitation laser beam relative to the applied in-plane transverse direct current.

The sample studied here is a δ-modulation-doped InAs quantum well [Fig. 1(a)]. The sample is cleaved along the 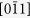 and 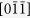 (denoted as the *x* and *y* directions, respectively) into a square of 3.5 mm × 3.5 mm, with two pairs of ohmic contacts 2 mm apart along the *x* and *y* directions, respectively. The ohmic electrodes are made by indium deposition. An in-plane transverse DC current is applied along the *x*-direction by the two indium electrodes, the photocurrent flowing along the *y-*direction is collected by another two indium electrodes. Linearly polarised light is passed through a rotatable quarter-wave plate, so that the sample is illuminated using elliptically polarised light with a circular polarisation degree defined by 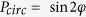, where *φ* is the angle between the polarisation plane of the laser radiation and the optical axis of the quarter-wave plate. The excitation beam is centred at the wavelength of 950 nm, with a diameter of 1.5 mm and an average power of about 10 mW, and irradiates the sample center obliquely, with incident angle of *θ* = 30° relative to the normal of the sample surface [Fig. 1(b)].

### HDPC by varying the amplitude of the transverse current

**Figure 2:**
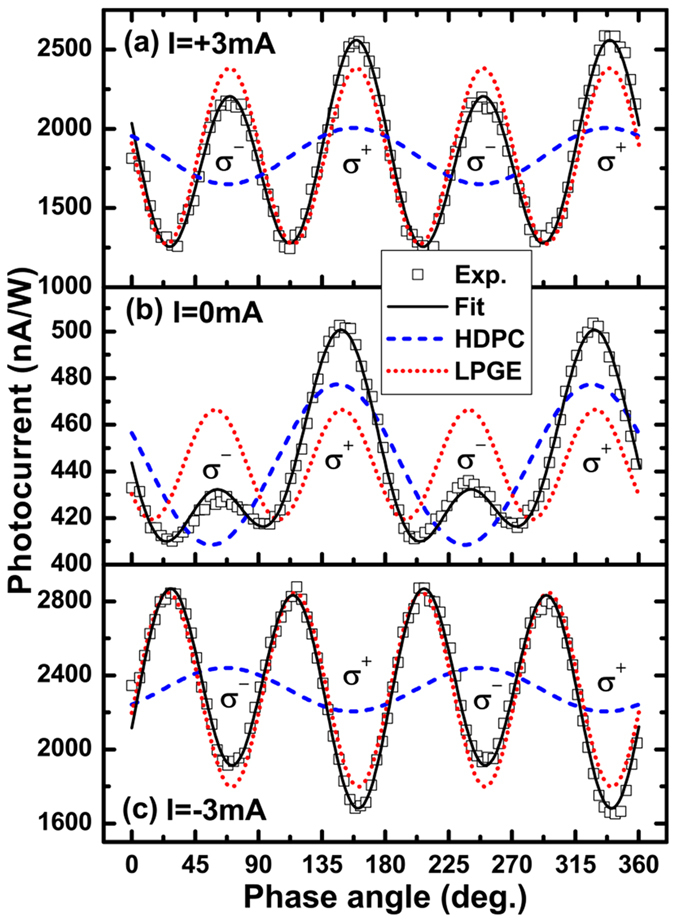
Typical photocurrent response at *β* = 0° (open squares) measured under the driving transverse current: (**a**) +3 mA; (**b**) 0 mA; (**c**) −3 mA. The fitted curves (solid lines) using Eq. (1) enable the overall photocurrent to be separated into its HDPC (blue dashed line) and LPGE (red dotted line) components.

**Figure 3:**
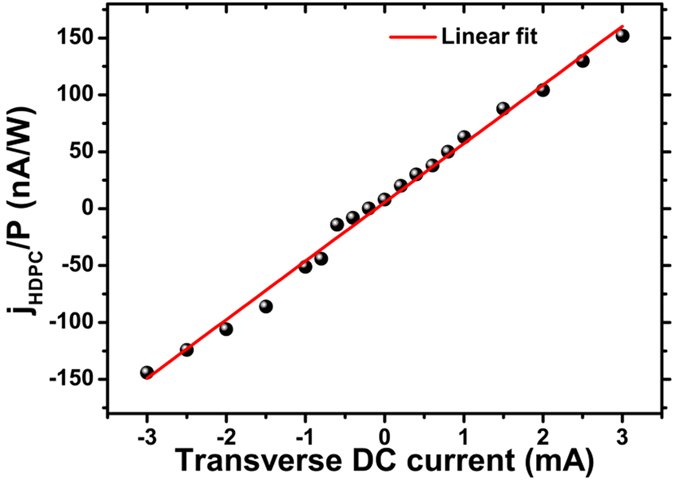
Dependence of HDPC on the transverse current (black dots) at *β* = 0° and its linear fit (red line).

The HDPC is first measured by varying the amplitude of the transverse current with the orientation of the incident plane of the excitation laser beam fixed at *β* = 0° (the orientation of the incident plane of excitation laser beam relative to the direction of the applied in-plane direct current is defined as the incident azimuth angle *β*) [Fig. 1(b)]. From the typical response of the photocurrent as a function of phase angle *φ* [Fig. 2], the photogalvanic current signal, consisting of two components with different periodicities, can be fitted using a formula similar to that for the photocurrent induced by the CPGE^9^,^10^,


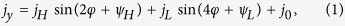


where *j*_*H*_ and *j*_*L*_ are the amplitudes of the HDPC, and the current induced by the linear galvanic effect (LPGE), respectively; *j*_*0*_ is the background current, which is independent of the polarisation state of the pumping light, and induced supposedly by the Dember effect^9^; *ψ*_*H*_ and *ψ*_*L*_ are the initial phases of the HDPC and the current induced by the LPGE, respectively. In a previous study^9^, the LPGE term has been attributed to the asymmetric scattering of free carriers in the non-centrosymmetrical system, and is not discussed in detail in this paper. We mainly focus on the HDPC component, which is actually the photocurrent induced by the CPGE when there is no transverse current applied. The typical response of the photocurrent (Fig. 2) is well fitted by Eq. (1); note the HPDC and LPGE contributions are also separated. The amplitudes of the HDPC measured at different transverse currents (Fig. 3) have been extracted; specifically the CPGE-induced photocurrent (namely the HDPC with no transverse current applied) has been subtracted to study the HDPC induced by transverse DC current only. The extracted amplitudes of the HDPC show a clear linear dependence on the transverse current with the HDPC reversing its sign when the current changes its direction.

### HDPC by varying the orientation of the excitation incident plane relative to the direction of the applied DC

**Figure 4:**
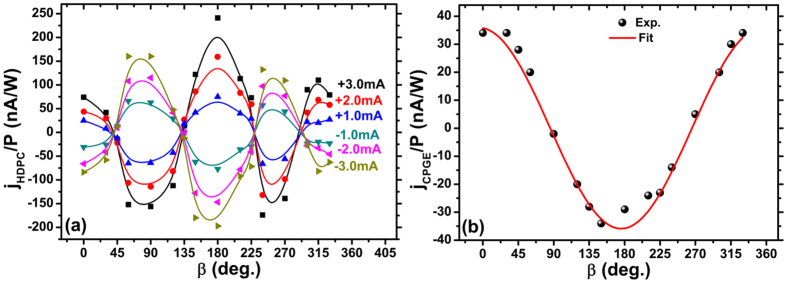
Incident azimuth angle dependence of: (**a**) the HDPC and (**b**) CPGE photocurrent, under different transverse currents.

**Figure 5:**
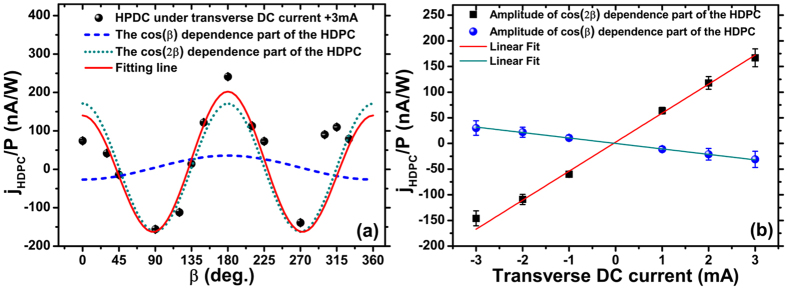
(**a**) Typical incident azimuth angle dependence of HDPC under the transverse current of +3 mA (black circle), the fitted curve (solid red line) obtained using equation (2), separates into its two components with cos(*β*) (blue dashed line) and cos(2*β*) (dark cyan dotted line) dependence; (**b**) Dependence of the amplitude of cos(*β*)- and cos(2*β*)-dependent components on the transverse current (blue circle and black square) and their linear fits (dark cyan and red lines).

To investigate further the effect of the transverse current on the HDPC, the dependence on incident azimuth angle (referred to as the *β* dependence) of the HDPC driven by different transverse currents is measured [Fig. 4(a); the CPGE-induced photocurrent has also been subtracted]. A strong *β* dependence is exhibited that is quite distinct from the purely CPGE-induced photocurrent, which has a cos(*β*) dependence [see Fig. 4(b)]. The maximum HDPC appears when the excitation incident plane is perpendicular or parallel (including anti-parallel) to the direction of the transverse current. The HDPC exhibits an approximate cos (2*β*) dependence. However, the HDPC at *β* = 0° is obviously different from that of *β* = 180°, which indicates that there probably exists an additional cos(*β*) dependence. Therefore, the *β* dependence of HDPC is fitted using


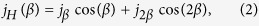


where *j*_*β*_ and *j*_*2β*_ are the amplitudes of the HDPC components proportion to cos(*β*) and cos(2*β*), respectively. From typical fitting results [Fig. 5(a)], contributions from the two terms of the HDPC were separated. The extracted amplitudes of *j*_*β*_ and *j*_*2β*_ for different transverse currents [Fig. 5(b)] also show a linear dependence on the transverse current.

In accordance with a previous report^24^, the *β* dependence of the CPGE photocurrent [Fig. 4(b)] can be described by


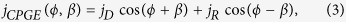


where *j*_*D*_ and *j*_*R*_ are the CPGE photocurrent components proportional to the Rashba and Dresselhaus constants, respectively; *ϕ* is the angle between the detected direction of the photocurrent and the 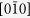 crystallographic axis. Our experimental data can be well fitted by Eq. (3) [Fig. 4(b)], indicating concordance with the result of previous work^24^. As the CPGE photocurrent has a cos(*β*) dependence, the component of the DC-induced HDPC can be considered as a modulation of the CPGE photocurrent by the transverse current.

## Discussion

**Figure 6:**
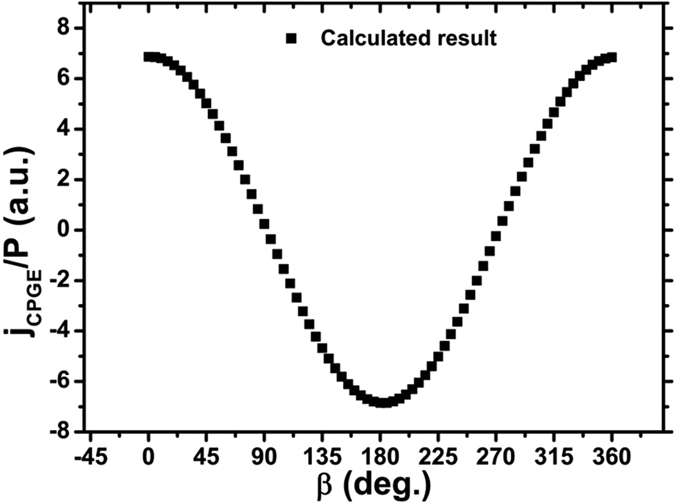
Calculated result of the incident azimuth angle dependence of the HDPC with no transverse current applied (the CPGE photocurrent).

To understand the complex *β* dependence of the observed HDPC, a theoretical model similar to that of refs 21 and 26 is adopted to calculate the CPGE photocurrent, then the influence of the transverse current is analysed. In our model, a 14-band **k•p** model is used to calculate the energy bands of the designed InAs quantum well structure. The photoexcited carrier density is determined within the density matrix formalism. The electric photocurrent *j*_*y*_ can then be calculated with 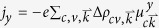, by summing the velocities of the photoexcited carriers. Here, 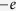 is the electron charge, *c* and *v* signify the conduction band and valence band, respectively; 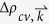 is the photoexcited carrier density distribution, and 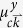 is the velocity of state 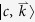 along the *y*-axis. The velocity 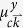 can be determined from 
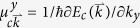
. In the relaxation time approximation, with direct optical transitions, the photoexcited carrier density distribution 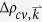 can be expressed as^9^:


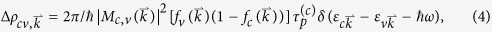


where 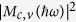 is the square of the optical transition matrix element, 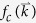 and 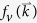 are the electron distribution function in the conduction and valence bands, respectively, and 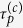 is the relaxation time in the conduction band. For simplicity, it is assumed that only the photoexcited electrons in the conduction band contribute to the photocurrent, and the contribution from the photoexcited holes is neglected. Furthermore, the photoexcited electron density distribution 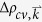 is considered to be related to the optical transition process only, with all the carrier relaxation processes neglected, so the relaxation time 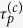 in Eq. (4) is treated as a constant. The CPGE photocurrent, *i.e.*, the *β* dependence of the HDPC without applying the transverse current, is calculated (Fig. 6) and reproduces our experimental observations of the cos(*β*) dependence of HDPC [Fig. 4(b)]. From Eq. (4), the photoexcited electron density distribution 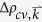 is proportional to the square of the optical transition matrix elements 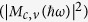, the distribution function 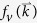 of the occupied state (the initial state of the optical transition process) in the valence band, and the distribution function 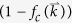 of the unoccupied state (final state of the optical transition process) in the conduction band. Once the in-plane transverse current is applied, the optical transition matrix elements remain constant because the applied direct current in our experiment is very small. The transverse current nevertheless creates an asymmetrical carrier distribution in both the conduction and valence bands^27^ (*i.e.*, it modifies the distribution functions of the initial and final states of the interband transition process). Consequently the photoexcited electron density distribution 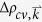 therefore changes through optical transitions, and the resulting photocurrent response is modulated. Because the conduction band of our InAs quantum well is isotropic, the HDPC response resulting from the asymmetry carrier distribution in the conduction band does not exhibit a more complex *β* dependence other than cos(*β*), as compared with the CPGE photocurrent. However, there exists a complex anisotropy associated with the valence band itself, even without DC current applied. Hence, the asymmetrical carrier distribution in the valence band induced by the transverse current should lead to a much more complex anisotropy for photoexcited electron density distribution 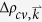. Therefore, the additional cos(2*β*) dependent response of the measured HDPC is attributed to a non-negligible photocurrent contribution from the asymmetrical carrier density distribution in the valence band caused by the transverse current. However, calculating the photocurrent contributed from such asymmetrical carrier distribution is much more complex when the valence bands are involved and beyond the scope of the present study.

In conclusion, a HDPC induced by the transverse current has been observed in an InAs quantum well; a linear dependence on transverse current is observed. Moreover, the HDPC exhibits different behavior with respect to the incident azimuth angle from that of the purely CPGE-induced photocurrent. A theoretical model is employed to calculate the HDPC at zero transverse current, and the effect of transverse DC current on the HDPC is analyzed. This HDPC appears to originate from an asymmetrical carrier distribution induced by the transverse current in both the conduction and valence bands that changes the photoexcited carrier density distribution through interband transitions. The observed efficient generation of the HDPC by the applied direct current offers an effective approach to electrically manipulate spin-related photocurrents in two-dimensional semiconductors for spintronic applications.

## Sample and Method

### Sample growth and characterisation

The sample studied here is a δ-modulation doped InAs quantum well grown on (100)-oriented semi-insulating GaAs substrate by molecular beam epitaxy. After depositing a 200-nm GaAs and 700-nm Al_0.7_Ga_0.3_Sb buffer layer, a bottom barrier of 50-nm AlSb, channel layer of 15-nm InAs, and spacer layer of 5-nm AlSb are deposited followed by a δ-doping layer of 1.5-nm InAs, respectively. Next a top barrier of 7.5-nm AlSb, 5-nm In_0.5_Al_0.5_As etch stop layer, and then a 5-nm cap layer are deposited on the top. The doping layer and cap layer are Si-doped to a doping concentration of ∼10^18^ cm^−3^. The sheet density of free electron is estimated to be 9.38 × 10^12^ cm^−2^, and the mobility, as measured using the Hall effect, is about 3.5 × 10^3^ cm^2^V^−1^s^−1^ at room temperature.

### Photocurrent measurement

The source of excitation light used in our photocurrent measurement is a pulsed Ti:sapphire laser providing ∼150-fs pulses with a repetition rate of 80 MHz. The wavelength of the pulsed laser can be tuned from 680 to 1080 nm. The photocurrent is collected by a current pre-amplifier and lock-in amplifier, combined with a chopper applied in the excitation laser beam. All measurements were performed at room temperature.

## Additional Information

**How to cite this article**: Li, J. B. *et al*. Helicity-dependent photocurrent induced by the in-plane transverse electric current in an InAs quantum well. *Sci. Rep.* **6**, 31189; doi: 10.1038/srep31189 (2016).
